# Beliefs and Risk Perceptions About COVID-19: Evidence From Two Successive French Representative Surveys During Lockdown

**DOI:** 10.3389/fpsyg.2021.619145

**Published:** 2021-02-01

**Authors:** Arthur E. Attema, Olivier L’Haridon, Jocelyn Raude, Valérie Seror, The COCONEL Group, Patrick Peretti-Watel

**Affiliations:** Aix Marseille Université, IRD, AP-HM, SSA, VITROME, Marseille, France (PP-W, VS, SC, CA, and LF); Southeastern Health Regional Observatory (ORS Paca), Marseille, France (PP-W, PV, and LF); Inserm CIC 1417, Université Paris, Faculté de Médecine Paris Descartes, AP-HP, Hôpital Cochin, Paris, France (OL); EHESP School of Public Health, Rennes, France (JR); CESP, Université Paris Sud, Faculté de Médecine UVSQ, Inserm, Université Paris-Saclay, Villejuif, France (FB and SL); Université Rennes, CNRS, CREM UMR 6211, Rennes, France (OH); and GEMASS, CNRS, Université Paris Sorbonne, Paris, France (JW).; ^1^EsCHER, Erasmus School of Health Policy & Management, Erasmus University, Rotterdam, Netherlands; ^2^Faculty of Economics, University of Rennes 1, Rennes, France; ^3^EHESP School of Public Health, Rennes, France; ^4^VITROME, Aix Marseille Université, IRD, AP-HM, SSA, Marseille, France

**Keywords:** beliefs, COVID-19, comparative optimism, rational learning, risk perception

## Abstract

**Background:**

The outbreak of COVID-19 has been a major interrupting event, challenging how societies and individuals deal with risk. An essential determinant of the virus’ spread is a series of individual decisions, such as wearing face masks in public space. Those decisions depend on trade-offs between costs (or benefits) and risks, and beliefs are key to explain these.

**Methods:**

We elicit beliefs about the COVID-19 pandemic during lockdown in France by means of surveys asking French citizens about their belief of the infection fatality ratio (IFR) for COVID-19, own risk to catch the disease, risk as perceived by others, and expected prevalence rate. Those self-assessments were measured twice during lockdown: about 2 weeks after lockdown started and about 2 weeks before lockdown ended. We also measured the quality of these beliefs with respect to available evidence at the time of the surveys, allowing us to assess the calibration of beliefs based on risk-related socio-demographics. Finally, comparing own risk to expected prevalence rates in the two successive surveys provides a dynamic view of comparative optimism with respect to the disease.

**Results:**

The risk perceptions are rather high in absolute terms and they increased between the two surveys. We found no evidence for an impact of personal experience with COVID-19 on beliefs and lower risk perceptions of the IFR when someone in the respondent’s family has been diagnosed with a disease. Answers to survey 1 confirmed this pattern with a clear indication that respondents were optimistic about their chances to catch COVID-19. However, in survey 2, respondents revealed comparative pessimism.

**Conclusion:**

The results show that respondents overestimated the probabilities to catch or die from COVID-19, which is not unusual and does not necessarily reflect a strong deviation from rational behavior. While a rational model explains why the own risk to catch COVID-19 rose between the two surveys, it does not explain why the subjective assessment of the IFR remained stable. The comparative pessimism in survey 2 was likely due to a concomitant increase in the respondents’ perceived chances to catch the disease and a decreased expected prevalence rate.

## Introduction

The outbreak of COVID-19 has been a major interrupting event for economies all over the globe. This event challenged the way societies and individuals deal with risks. Over and above public policies, an essential determinant of the spread of the virus is a series of small-scale individual decisions, such as wearing face masks in public space, regularly washing hands or deciding how often to go the office, class, store or anywhere else. According to decision theory, those decisions depend on tradeoffs between costs (or benefits) and risks, and those tradeoffs are deeply rooted in individual preferences. Considering the wide literature that has been devoted to the understanding of individual preferences and attitudes toward risk, their heterogeneity and their determinants (Dohmen et al., 2011), one of the key figures of the classical representation of behavior under uncertainty is that beliefs - in addition to risk attitudes - are key to explain decisions whenever they involve money, life duration, health states, approval of friends or well-being of others (Savage, 1954).

Uncertainty especially affects decisions regarding health where most probabilities are ambiguous and not objectively known (Attema et al., 2018). Smoking is a typical example of decisions for which subjective assessments of mortality risks have received a lot of attention (Viscusi, 1990; Khwaja et al., 2007). Beliefs and, more generally, risk perceptions are rather challenging to evaluate, especially for a new disease such as COVID-19. First, risk perceptions are threat-specific most of the time and incorporate different kinds of information through deliberative, affective and experiential processes (Slovic et al., 2004; Ferrer and Klein, 2015). In case of a new disease, the amount of available information, whenever it is publicized numerically or derived from personal experience, is limited. Second, the range of available methods to elicit beliefs is restricted. Experimental studies, which measure beliefs with monetary stakes, offer an array of incentive-compatible elicitation methods (Trautmann et al., 2015), but these methods are known to be difficult to implement in large representative samples (Dohmen et al., 2011). Additionally, incentive compatibility has no bite for events with serious health consequences such as COVID-19. In such cases, survey studies to assess beliefs and risks generally involve introspective judgments to assess beliefs and risks (Viscusi, 1990; Coe et al., 2012; Carman and Kooreman, 2014). Third, there is no pre-existing measuring rod to judge the accuracy of risk perceptions. In particular, without objective and subjective benchmarks, it is difficult to know if a low risk perception to catch the disease actually reflects optimism or pessimism, whenever it is considered as an absolute or a relative measure (Shepperd et al., 2013). Thus, understanding, predicting and aiding individual decisions in face of COVID-19 mainly relies on answers on a series of questions (Fischhoff, 2020), such as “how much of the disease is in the community?” (i.e., beliefs about the prevalence rate), “what is my risk of exposure to the COVID-19?” or “what is the risk to die if infected?” [i.e., beliefs about the infection fatality ratio (IFR)]. Because individual decisions are likely to be impacted by others’ decisions, and therefore by their beliefs, second-order beliefs (“how do the others perceive the risk?”) might also be of importance.

Several recent studies have reported information on the disease risk perception of COVID-19, and its perceived impact on health during the lockdown phase due to COVID-19, especially in Italy where the virus reached Europe the earliest, but also in other countries [e.g., (Barattucci et al., 2020; Cerami et al., 2020; Faasse and Newby, 2020; Lanciano et al., 2020; Liu M. et al., 2020; Moyce et al., 2020)]. These studies have shown that people perceive the impact of COVID-19 on their (mental) health as high (Tull et al., 2020; Xiong et al., 2020), and that their risk perception of this disease is correlated with adoption of preventative health behaviors (Dryhurst et al., 2020), but its level is not so high (Commodari and La Rosa, 2020; Lanciano et al., 2020; Liu M. et al., 2020), and lower than their concern for the future and for economic and social consequences of the pandemic (Lanciano et al., 2020). In this paper, we add to this literature by investigating beliefs about the COVID-19 pandemic during lockdown in France, analyzing responses to survey items that ask for the individuals’ judgments of the risks associated with COVID-19. Self-assessments about risks included the IFR for COVID-19, the surveyed individuals’ own personal risk to catch (or catch again) the disease, the risk as perceived by others and, finally, the expected prevalence rate after the COVID-19 pandemic in France. Those four self-assessments were measured twice during lockdown. In addition to shedding light on the determinants of beliefs in a representative population sample and their dynamics during lockdown, the paper also provides measures of the quality of these beliefs with respect to available evidence at the time of the surveys. Finally, comparing own personal risk to catch COVID-19 to expected prevalence in the two successive surveys provided a dynamic view of comparative optimism with respect to the disease. The organization of the paper is as follows. The next Section introduces the theoretical background and our resulting hypotheses. The Section ‘Materials and Methods’ describes the data from the two successive surveys and how we computed measures of objective risks. ‘Results’ presents the statistical methods used to analyze the survey responses and investigates dynamics, heterogeneity and determinants of the self-assessment of beliefs, assesses the quality of beliefs through calibration. and reports the dynamics of comparative optimism during lockdown. Finally, the last Section discusses the results and concludes.

## Theoretical Background and Hypotheses

The Bayesian learning model (Viscusi, 1991) assumes individuals have three risk information sources, with each source characterized by its informational content. The first source is the prior risk assessment, a fundamental element of any Bayesian model. In the absence of information, a natural prior is the uninformative prior. The second source is the experience of the individual. Experience regroups direct individual experience with the risk, such as catching COVID-19 and indirect experience, e.g., knowing a close family/friend ill from COVID-19. Lockdown was used as an essential element of personal experience. During lockdown, limited physical and social interactions reduced the number of observed cases of infection from COVID-19, as well as observed fatalities. Experience predicts a decrease in the perceived risks to catch COVID-19, expected prevalence or lethality of the disease during lockdown. Because limited social interactions reduced the exchanges of private information upon beliefs, restricted personal experience with others’ beliefs predicts stability of these beliefs during lockdown. The third source of information is the risk information provided to the individual. Risk information includes public information about the risk and observable events processed as information by the individual. Typical sources of risk information are media coverage or public messages from the authorities. In the case of COVID-19, existing medical evidence covered by media identified age, pre-existing chronic illness, and -to a lesser extent- gender as risk factors for the severity and lethality of the disease. Location was an important risk information for the localization of the disease. Publicized information on the epidemic dynamics, with a peak occurring in the middle of lockdown, was another source of indirect risk information for individuals. A formal elaboration of this model can be found in the Supplementary Material.

Apart from the Bayesian learning model, several insights from psychology might help formulating our hypotheses. First, comparative optimism refers to the phenomenon that people believe the probability that a future negative event happens to themselves is lower than the probability that it happens to a similar other person, and vice versa for positive events (Shepperd et al., 2013). Several empirical studies found evidence supporting comparative optimism in a health context (Weinstein, 1980; Hoorens, 1994; Shepperd et al., 2013; Kuper-Smith et al., 2020). Second, the representativeness heuristic entails that someone evaluates a subjective probability by the degree of correspondence between the sample and the population (Kahneman and Tversky, 1972; Tversky and Kahneman, 1974). As such, it emphasizes the generic features of an event. This heuristic has also been observed frequently in health settings (Attanasio et al., 1998; Brannon and Carson, 2003), for example in the long-lasting belief of medical experts that ulcers were caused by stress, while in fact they are caused by bacteria (Gilovich and Savitsky, 2002). Finally, the familiarity bias stems from the availability heuristic and holds that events are judged as more frequent or more important if they are more familiar in memory (Tversky and Kahneman, 1974). Evidence of this bias in health was reported by Sherman et al. (1985) and Pachur et al. (2012).

Consistent with the Bayesian learning model and the summarized literature, this study aims to test the following hypotheses. First, we test if people have well-calibrated (i.e., accurate) beliefs about the prevalence rate, their probability of getting infected with COVID-19, and the IFR, and if these are mediated by socio-demographic characteristics such as gender and age (Weinstein and Klein, 1995; de Zwart et al., 2009; Vaughan, 2011; Clifton et al., 2016; Moran and Del Valle, 2016; Clark et al., 2020; Raude et al., 2020) (H1). It could also be that the lockdown has decreased risk perceptions, because people have the feeling that lockdown helps control, which in turn lowers risk perceptions (Nordgren et al., 2007). Second, we test comparative optimism by comparing respondents’ beliefs about these probabilities for themselves and for others (H2). The Bayesian model could justify this if beliefs reflect perfect ignorance at the beginning of the pandemic, since as people gather more information on their close environment, they should update their own beliefs (downward) more than their beliefs for the other, for which they gather less information. In addition, the familiarity bias suggests people might be excessively optimistic about their own beliefs (Kilka and Weber, 2000; Heideker and Steul-Fischer, 2015). Third, we test if these beliefs change during the pandemic by comparing the results from survey 1 and survey 2 (H3). The Bayesian model and the representativeness heuristic might differ here. While the Bayesian model predicts that individuals rationally update their beliefs based on new information obtained during the lockdown, the representativeness heuristic predicts individuals to underweight new information and stick to their prior risk assessment despite new evidence.

## Participants and Methods

### Participants

Between March 31 and April 27, 2020, we conducted surveys in two representative samples of the French population 18 years of age and over (*n* = 1,005 and *n* = 1,004). The study design was approved by the ethical committee of the University Hospital Institute Méditerranée Infection (#2020-018). Participants to the surveys were selected and interviewed by IFOP (Paris, France), a survey research company. Participants were drawn from an online research panel of more than 750 000 nationally representative households of the French general population, that is developed and maintained by IFOP. Data were collected using an online survey between March 31 and April 2 for the first survey and between April 23 and April 27 for the second survey. The first survey was conducted two weeks after the nationwide lockdown was introduced (lockdown was active between March 17 and May 11, 2020) and the second survey was conducted two weeks before the lockdown ended. Internal procedures at IFOP explain the small difference in the number of participants between the two surveys (*n* = 1,005 vs. *n* = 1,004).

Prior information on the panelists was used to determine eligibility and to draw a random sample, stratified to match French official census statistics for sex, age, occupational status, education level, size of town, and region. Table 1 provides details about the socio-demographic characteristics of the participants and health status.

**TABLE 1:** Socio-demographic characteristics.

**Characteristics**		**Survey 1 (N = 1005)**	**Survey 2 (N = 1004)**
Gender	- Female	527 (52.4%)	526 (52.4%)
	- Male	478 (47.6%)	478 (47.6%)
Age category	- less than 19	23 (2.3%)	27 (2.7%)
	- 20–29	122 (12.1%)	144 (14.3%)
	- 30–39	190 (19.0%)	155 (15.5%)
	- 40–49	174 (17.3%)	182 (18.2%)
	- 50–59	185 (18.4%)	188 (18.7%)
	- 60–69	140 (13.9%)	135 (13.5%)
	- 70+	171 (17.0%)	172 (17.1%)
Marital status	- Single	319 (31.7%)	335 (33.3%)
	- Live in couple	686 (68.3%)	669 (66.7%)
Education	-<high school	513 (51.0%)	509 (50.7%)
	- ≥bachelor degree	155 (15.4%)	159 (15.8%)
	- high school or<bachelor degree	337 (33.6%)	337 (33.5%)
Labor market status	- Inactive	419 (41.7%)	407 (40.6%)
	- Employe, private sector	353 (35.1%)	360 (35.9%)
	- Employe, public sector	103 (10.2%)	92 (9.1%)
	- Self-employment	55 (5.5%)	47 (4.7%)
	- Unemployed	75 (7.5%)	97 (9.7%)
Income	- N-Missing answers	122	115
	- Low income	341 (38.6%)	365 (41.1%)
	- High income	130 (14.7%)	141 (15.8%)
	- Middle income	412 (46.7%)	383 (43.1%)

### Measures

The questionnaire collected data on socio-demographics (Table 1), self-assessments of risks about COVID-19, confidence in beliefs, opinions toward COVID-19 and seasonal influenza, personal information about COVID-19 and health characteristics. Other questions relating to habits during lockdown and attitudes toward vaccination were included in both surveys, while questions related to sleep problems (survey 1), cultural profiles (survey 2) and specific medical treatments (survey 2) were included in one of the two surveys. Those questions are not included in the current analysis, but the interested reader on these topics is referred to The COCONEL Group (2020) for a description of items and responses.

#### Quantitative Measures: Assessment of Risks About COVID-19 and Seasonal Influenza

We measured self-assessments of risks about COVID-19 with four items. The first item concerned the perception of the IFR for COVID-19 (Q1: “Out of 100 people who are infected with the Coronavirus [COVID-19], how many of them die from the disease?”). Literature on smoking risk perception suggests that a base population reference point is a more readily understood method for eliciting probabilistic information about death rates than explicitly dealing with probabilities or fractions (Viscusi and Evans, 1990). Survey 1 included a replication of question Q1 for seasonal influenza (Q1bis: “Out of 100 people who are infected with seasonal influenza, how many die from the disease?”). In order to avoid order effects between items Q1 and Q1bis, their order was randomized for each respondent.

The second item was an assessment of own personal risk to catch -or catch again in case the respondent has already been ill from the Coronavirus [Q2: “What risk do you have [to catch/to catch again] the Coronavirus [COVID-19] by the end of the epidemic (on a 0–100 scale)?”]. Third, we assessed how other people perceive their risk to catch the disease [Q3: “How do the French generally assess their risk of catching the Coronavirus [COVID-19] by the end of the epidemic (on a 0–100 scale)?”]. For items Q2 and Q3, we chose a numerical format rather than a purely qualitative answer scale because the former allows more variability than the latter. It is also generally associated with better prediction of behaviors (Juster, 1966; De Bruin et al., 2011). The fourth item corresponded to the expected prevalence of COVID-19 in the French population [Q4: “By the end of the epidemic, what do you think will be the proportion of the French population who have had the Coronavirus [COVID-19] (on a 0–100 scale)?”].

#### Confidence in Beliefs

Survey 1 included two questions about confidence in beliefs for items Q2 and Q3 (“How confident do you feel in your answer?”: a. very high, b. high, c. moderate, d. low, e. very low”).

#### Qualitative Measures: Opinions Toward COVID-19

We evaluated opinion toward COVID-19 with numerical 11-point scales for which participants were asked to give a score between 0 and 10. Those included relative risk (Q5: “Compared to other French people how would you rate your own risk of catching the Coronavirus [COVID-19]? Give a score between 0 and 10: the score 0 indicates that you think you are much less at risk than other French people and the score 10 that you think you are much more at risk than other French people. The intermediate notes allow you to qualify your judgment.”), concern about the disease (Q6: “Are you worried about [catching/catching again] the Coronavirus [COVID-19] by the end of the epidemic? Give a score between 0 and 10: a score of 0 means that you are not at all worried about the possibility of [catching/catching again] the Coronavirus (COVID-19) at all, and a score of 10 means that you are very concerned. The intermediate notes allow you to qualify your judgment.”), contagiousness (Q7: “how contagious is the Coronavirus [COVID-19]? Give a score between 0 and 10, with a score of 0 indicating that this disease is not very contagious and a score of 10 that it is very, very contagious. The intermediate notes allow you to qualify your judgment.”) and seriousness (Q8: “how serious is the COVID-19? Give a score between 0 and 10: a 0 indicates that catching this disease is not at all serious and a 10 that it is very, very serious. The intermediate notes allow you to qualify your judgment.”) of the disease. Survey 1 also included replications of items Q7 (contagiousness: Q7bis) and Q8 (seriousness: Q8bis) for seasonal influenza.

#### Health Related Items Regarding the Pandemic and Health Status

Specific items regarding the pandemic included whether participants had been diagnosed with -or ill from- COVID-19 and whether some of their relatives (family members or friends) had been infected. Participants were also asked to provide an expectation of the duration of the pandemic (in weeks on a 0-52 scale: “When do you think this epidemic will be truly over?”). In addition to socio-demographic characteristics, the first survey also included an item on self-reported general health status (“In general, how would you rate your state of health? Very good, fairly good, bad, quite bad”) and an item related to chronic illness (“Do you suffer from a chronic, that is to say long-lasting, disease or health problem that requires medical attention (for example: diabetes, heart or respiratory disease)? Disregard temporary or temporary health problems, such as the flu.”).

### External Data Sources

**FIGURE 1:**
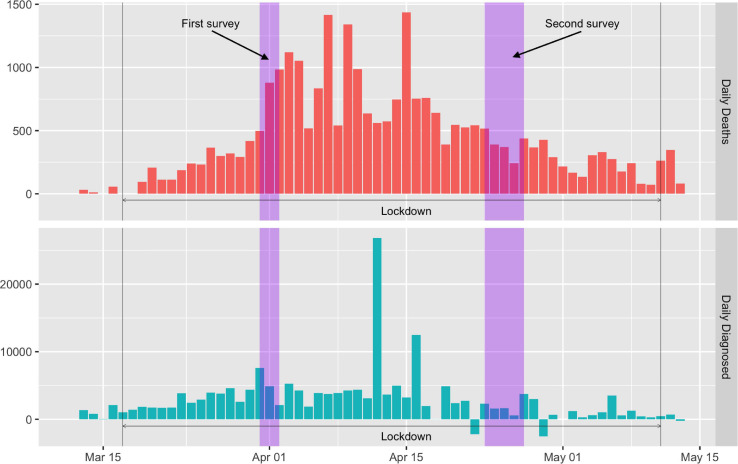
The two surveys during French national lockdown.

We also collected the available data on the number of recorded diagnoses with COVID-19 and deaths from COVID-19 in hospital in France at the time of survey administration. Those data, the only ones recorded for COVID-19 at the time of the two surveys, are publicly available from Santé Publique France, the national public health agency (https://www.santepubliquefrance.fr/). Figure 1 shows a timeline of the lockdown in France alongside the two surveys and the number of daily deaths recorded in hospital (top panel) and the number of daily cases diagnosed in hospital (bottom panel). Figure 1 also shows that the epidemic peak occurred between the two surveys, both for daily deaths (top histogram) and for diagnosed cases (bottom histogram).

We considered two datasets. The first dataset includes the number of deaths and diagnosed cases by gender and administrative French metropolitan regions. The second dataset also includes the number of deaths and diagnosed cases but aggregated by age category and “department” (a sub-division of administrative regions). To obtain a consistent measure of location areas between the two datasets, we merged departments into administrative regions in the second dataset to obtain a dataset by age category and region. We fixed the date at the center of the time interval within each survey. We computed the IFR as the ratio of the deceased persons over the number of diagnosed cases.

Because these data are hospital data only and in a context where confirmations of COVID-19 infections mostly occurred when being hospitalized, the diagnosed cases corresponded to the most severe cases of the disease, leading to a computed IFR that can be thought as upper bounds of the effective IFR. To translate the number of diagnosed cases into population proportions to measure prevalence, we collected population data on January 1, 2020 on gender, age and location area from the INSEE (the French national institute of statistics). Still, with data being restricted to hospital reports only and recorded at the beginning of the epidemic, the recorded cases corresponded to the most severe cases of the disease. A consequence is that the computed prevalence rate can be thought as a lower-bound of the population risk to catch COVID-19.

Table 2 shows the computed IFR and prevalence rate by gender, age category and region. For the sake of clarity, and following Salje et al. (2020), we categorized regions into three geographical main areas in France by importance of incidence: highest incidence regions (Paris region and north-eastern part of the country; namely, Île-de-France and Grand-Est), medium incidence regions (located in the center-east part of the country: Hauts de France, Bourgogne-Franche-Comté, Auverge-Rhône-Alpes, Corsica, Provence-Alpes-Côte d’Azur and Center-Val-de-Loire) and lowest incidence regions (all located in the west part of the country: Normandie, Bretagne, Pays de la Loire, Nouvelle-Aquitaine and Occitanie). To provide a sensible view of the differences between categories, Table 2 reports the values for a reference case (a prototypical individual with the lowest risk: a woman, aged 20–29 in Nouvelle-Aquitaine region) and odd-ratios for alternative characteristics. Table 2 shows that COVID-19 was more prevalent among men, older people and people living either in the medium and highest incidence regions located in the north-east part of France (Hauts de France, Bourgogne-Franche-Comté and Grand-Est), Corsica (Corse) or the Paris area (Île de France). For example, Table 2 shows that the computed prevalence rates among the elderly was around 13 times higher than the prevalence rate among people aged 20-29 and their IFR was 40 to 50 times higher.

**TABLE 2:** Available data at the time of the surveys on population death ratios and diagnosed ratios by gender, age category and region.

**Socio-demographic variables**	**Category**	**prevalence, survey 1**	**prevalence, survey 2**	**IFR, survey 1**	**IFR, survey 2**
female (1/100)		0.047	0.101	9.343	16.141
odds-ratio	Male	1.589	1.270	1.084	1.240
20–29 (1/100)		0.014	0.027	0.391	0.782
odds-ratios	30–39	1.906	1.759	1.773	1.986
	40–49	3.283	2.851	2.982	3.281
	50-59	5.869	4.830	6.086	6.965
	60–69	8.297	6.793	12.100	13.930
	70+	12.594	13.366	49.832	40.453
Nouvelle-Aquitaine (1/100)		0.019	0.037	8.828	16.330
odds-ratios					
Lowest incidence regions	Bretagne	1.188	0.952	0.699	0.836
	Occitanie	1.379	1.371	0.950	0.974
	Normandie	1.506	1.394	0.842	1.128
	Pays de la Loire	1.587	1.323	0.697	0.762
Medium incidence regions	Provence-Alpes-Côte d’Azur	1.787	2.294	1.023	0.980
	Center Val-de-Loire	2.532	2.919	1.327	1.217
	Auvergne-Rhône-Alpes	2.869	2.845	0.519	0.719
	Hauts-de-France	3.004	2.612	0.878	0.955
	Corse	3.469	1.919	1.067	1.250
	Bourgogne-Franche-Comté	3.533	3.536	1.421	1.321
Highest incidence regions	Île-de-France	6.657	6.439	1.017	1.154
	Grand-Est	7.163	5.944	1.732	1.370

### Statistical Analysis

We measured the determinants of beliefs for each item Q1 to Q4 using a Generalized Linear Model with a logit link and a quasibinomial distribution. We explored the dynamics of beliefs by comparing answers to items Q1 to Q4 between the two surveys with a base GLM model with no covariates. A difficulty with self-assessment of beliefs on 0–100 scales is the usually high frequency of the answer “50” in the responses. This “50 blip” might be an important source of bias in belief measurement (de Bruin et al., 2002). To account for such a possible bias, we fitted a beta function to each distribution of items and subtracted the expected proportion of responses in the 45-55 category from the proportion actually observed to infer the number of excess 50s over those expected in the best fit distribution. In addition, we measured the significance of the difference between distributions with and without the “50” answers by regressing the demeaned answers to item Q1 to Q4 on an indicatrix of the “50” answer. We set statistical significance at *p* < 0.05.

We regressed the answers to items Q1-Q4 measured as proportions on the answers to socio-demographic items, health-related items, qualitative items related to COVID-19 and, when relevant, beliefs about the seasonal influenza. For each item, we reported the results of the regressions for both the first survey only and the two surveys pooled together. In order to ease interpretation of the regression coefficients, we report average marginal effects, with standard errors computed using the Delta method. All statistical analyses were performed using R software, version 3.7.0. The Generalized Linear Model regressions were based on the *survey* package, using post-stratification weights, and average marginal effects computed with the *margins* package. Estimates from the GLM regression with and without post-stratification weights are reported in Tables 6, 7 in the Supplementary Material.

We compared responses to items Q1–Q4 with available information at the date of the surveys to measure calibration of beliefs. We evaluated calibration by comparing the self-assessment of the IFR with the expected prevalence based on a paired t-test. Existing scientific evidence suggested that prevalence (the population risk to catch the disease) was expected to be higher than IFR (the population risk to die from the disease if infected), which justified the use of a one-sided test. We also measured calibration of beliefs based on the comparisons between the IFR from COVID-19 and from the seasonal influenza. This measure was possible in the first survey only, and statistical significance was assessed based on a (two-sided) paired t-test. In addition, a comparison of answers to questions on contagiousness and seriousness for illness-seasonal influenza vs. COVID-19 provided a qualitative evaluation of the consistency of answers. For both, we used a linear regression without intercept. For contagiousness we regressed the answer to question Q7 on the answer to question Q7bis to obtain a measure of the assessed difference in contagiousness between the two diseases. We then compared this measure with existing evidence at the time of the survey. Our last calibration exercise used available hospital data described in Section 2.3 to measure the difference between beliefs about own personal risk to catch COVID-19 (item Q2) and public information on prevalence rates by gender, age category and location area. Lastly, we measured comparative optimism during lockdown by comparing answers to item Q2, the own personal risk perception to catch COVID-19, and answers to item Q4, the perceived prevalence for COVID-19. Significance levels were assessed based on paired Student t-tests.

## Results

### Descriptive Statistics

**FIGURE 2:**
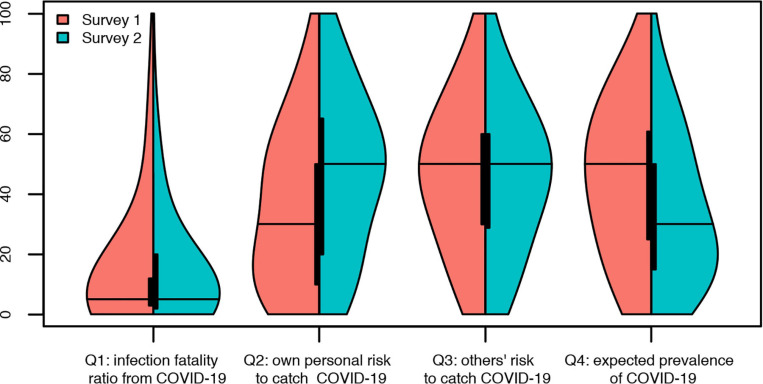
Distribution of answers to questions Q1 to Q4 between the two surveys.

Descriptive statistics are illustrated in Tables 1 and 3 and in Figure 2. Figure 2 shows the distribution of answers to items Q1 to Q4 for the two surveys. For each item, the left part of the violin plot shows the distribution of answers for the first survey and the right part of the violin shows the distribution of answers for the second survey.

**TABLE 3:** Descriptive statistics.

**Item**		**Survey 1 (N = 1005)**	**Survey 2 (N = 1004)**
Q1: IFR	- N-Missing answers	280	263
	- Mean (SD)	16.457 (22.221)	16.106 (19.266)
Q1bis: infection fatality ratio from seasonal influenza	- N-Missing answers	347	
	- Mean (SD)	12.654 (20.500)	
Q2: own risk to catch COVID-19	- N-Missing answers	291	274
	- Mean (SD)	34.481 (26.477)	45.940 (26.599)
Confidence in assessment of own risk to catch COVID-19	- N-Missing answers	291	
	- Very high	45 (6.3%)	
	- High	215 (30.1%)	
	- Moderate	385 (53.9%)	
	- Low	45 (6.3%)	
	- Very low	24 (3.4%)	
Q3: others’ risk to catch COVID-19	- N-Missing answers	373	381
	- Mean (SD)	47.853 (23.943)	46.205 (24.250)
Confidence in assessment of others’ risk to catch COVID-19	- N-Missing answers	373	
	- Very high	32 (5.0%)	
	- High	176 (27.8%)	
	- Moderate	351 (55.6%)	
	- Low	65 (10.2%)	
	- Very low	9 (1.4%)	
Q4: expected prevalence	- N-Missing answers	276	308
	- Mean (SD)	45.049 (25.058)	33.927 (23.707)
Expected duration (in weeks)	- Mean (SD)	13.048 (9.048)	31.023 (16.120)
Q5: relative risk to get COVID-19	- N-Missing answers	175	69
	- Mean (SD)	5.599 (2.371)	5.663 (2.466)
Q6: worried to get COVID-19	- N-Missing answers	58	39
	- Mean (SD)	6.055 (2.642)	6.348 (2.656)
Q7: contagiousness of COVID-19	- N-Missing answers	59	44
	- Mean (SD)	8.169 (1.860)	7.895 (2.021)
Q7bis: contagiousness of seasonal influenza	- N-Missing answers	96	
	- Mean (SD)	6.740 (1.980)	
Q8: seriousness of COVID-19	- N-Missing answers	39	51
	- Mean (SD)	8.152 (1.837)	7.863 (2.029)
Q8bis: seriousness of seasonal influenza	- N-Missing answers	75	
	- Mean (SD)	6.477 (2.040)	
Q5: relative risk to get COVID-19	- N-Missing answers	175	69
	- Mean (SD)	5.599 (2.371)	5.663 (2.466)
Q6: worried to get COVID-19	- N-Missing answers	58	39
	- Mean (SD)	6.055 (2.642)	6.348 (2.656)
Q7: contagiousness of COVID-19	- N-Missing answers	59	44
	- Mean (SD)	8.169 (1.860)	7.895 (2.021)
Q7bis: contagiousness of seasonal influenza	- N-Missing answers	96	
	- Mean (SD)	6.740 (1.980)	
Q8: seriousness of COVID-19	- N-Missing answers	39	51
	- Mean (SD)	8.152 (1.837)	7.863 (2.029)
Q8bis: seriousness of seasonal influenza	- N-Missing answers	75	
	- Mean (SD)	6.477 (2.040)	
General health status	- Very good	209 (20.8%)	
	- Good	659 (65.6%)	
	- Bad or very bad	137 (13.6%)	
Chronic illness	- N-Missing answers	30	
	- No	685 (70.3%)	
	- Yes	289 (29.7%)	
Has been diagnosed/ill from COVID-19	- No	994 (98.9%)	976 (97.2%)
	- Yes	11 (1.1%)	28 (2.8%)
Close family/friend ill from COVID-19	- No	761 (75.7%)	746 (74.3%)
	- Yes	244 (24.3%)	258 (25.7%)

### Calibration of Beliefs

Calibration of reported beliefs was assessed based on both the consistency of answers and their comparison with available information at the time of the surveys.

#### Infection Fatality Ratio vs. Prevalence Rate

We first evaluated calibration by comparing the self-assessment of the IFR with the expected prevalence. Available scientific evidence suggests that COVID-19 is highly contagious and only severe forms of the disease led to fatal issues. Notably, the prototypical case of the Diamond Princess cruise ship suggested an IFR at least ten times lower than the prevalence rate. On 3rd February 2020, an outbreak of COVID-19 was reported on the Diamond Princess cruise ship, with initially 10 persons confirmed to be infected with the virus. The outbreak of COVID-19 led 3711 crew and passengers to be quarantined for three weeks. By the end of February, 7 persons had died among the 705 persons diagnosed and tested positive, giving an IFR of 0.99% and an observed prevalence rate of 19%. The IFR from the Diamond Princess cruise ship was much lower than the values reported in March 2020, which were closer to 3-4% (Rocklöv et al., 2020).

The average IFR provided by the respondents was equal to 16.46 in survey 1 and to 16.1 in survey 2, around two to three times lower than the reported expected prevalence rate (45.05 in survey 1, 33.93 in survey 2). In both surveys the differences between the answers to the corresponding items Q1 and Q4 were highly significant (one-sided paired *t*-tests, both *P*-values < 0.01).

#### COVID-19 vs. Seasonal Influenza

**FIGURE 3:**
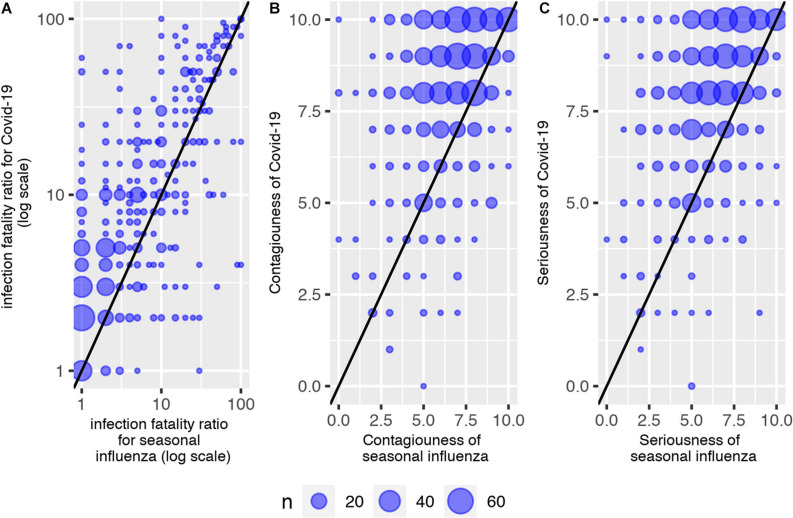
COVID-19 vs. seasonal influenza in survey 1. **(Panel A)**: IFR. **(Panel B)**: Contagiousness. **(Panel C)**: Seriousness.

Survey 1 contained a replication of the assessment of the IFR for the seasonal influenza. The number of deaths from COVID-19 during the first wave of the epidemic corresponded to at least twice the usual mortality from seasonal influenza (Équipes de surveillance de la grippe, 2019). Usual estimates of the IFR for seasonal influenza in France are less than 0.5%. As a source of comparison, Rajgor et al. (2020) reported a common IFR of 0.1% for influenza and - based on the above mentioned case of the Diamond Princess cruise ship - a rate of 1% for COVID-19. This is much less than the rough upper-bound measure for the IFR in France, according to the hospital data for severe cases of COVID-19: Table 1 shows that the available public information about COVID-19 corresponded to an IFR around 10% at the time of survey 1. Figure 3 shows the comparisons between assessments for COVID-19 and for seasonal influenza in survey 1. Figure 3-A shows that for most respondents (*n* = 455), the IFR of COVID-19 was higher than that of seasonal influenza. Otherwise, similar IFRs were reported by 103 respondents, whereas 97 reported lower rates. The difference between the two IFRs was highly significant (paired Student *t*-test, *P* value < 0.01).

Answers to qualitative questions on seriousness and contagiousness of seasonal influenza vs. COVID-19 confirmed the good calibration of beliefs about COVID-19. Figure 3 shows the distribution of answers to the qualitative question on contagiousness (panel B) and seriousness (panel C) for seasonal influenza (on the x-axis) and COVID-19 (on the y-axis). A linear regression without intercept for reported contagiousness showed that participants estimated contagiousness of COVID-19 to be 1.17 (standard error 0.01), i.e., higher for COVID-19 than for seasonal influenza. This is in line with available evidence on contagiousness at the time of survey 1. For example, Biggerstaff et al. (2014) report a median reproduction number (R0) for seasonal influenza to be around 1.3, while a review by Liu Y. et al. (2020) reports a median R0 for COVID-19 of 2.8, and evidence on the Diamond Princess cruise ship (Rocklöv et al., 2020) shows that isolation and quarantine lowered the R0 to 1.78 (1.37 higher than that of seasonal influenza). Additionally, a minority of *n* = 22 individuals provided qualitative answers to questions Q7, Q7bis, Q8, and Q8bis revealing they thought COVID-19 to be less serious and less contagious than seasonal influenza.

#### Calibration of Own Personal Risk to Catch COVID-19 and Socio-Demographic Characteristics

**FIGURE 4:**
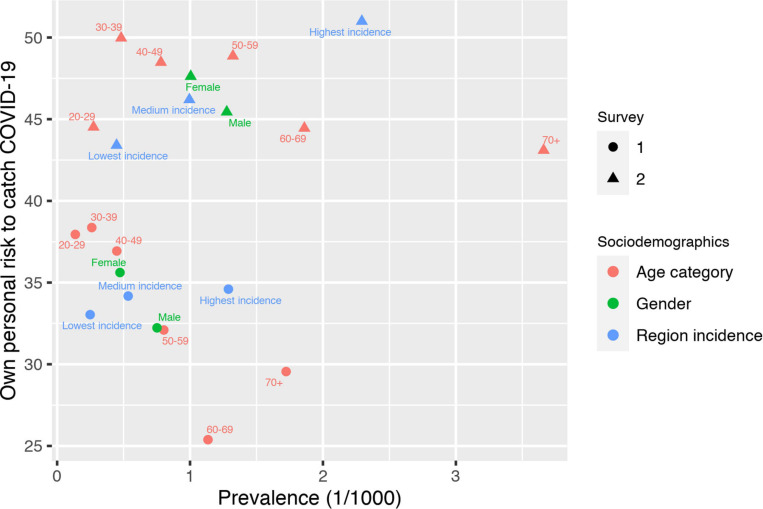
Own personal risk to catch COVID-19 and prevalence by sociodemographic categories.

Figure 4 shows the comparison of the average answers to question Q2 (own personal risk to catch COVID-19) with the available information on prevalence at the time of the surveys by gender, age category, location and survey date. For all sociodemographic groups, the own personal risk to catch COVID-19 increased between the two surveys. Own personal risk was well-calibrated with respect to location: it was lower in low-incidence regions and higher in high-incidence regions. Things were different for gender and age: women expressed higher risks than men, although the available scientific evidence systematically showed a different pattern. The same applied to age: younger people view themselves as being at higher risk than older people and this despite the rather large difference in prevalence between age classes.

Considered jointly, these findings are summarized in the following observation:

H1: Risk perceptions in absolute terms were rather high for the IFR, the expected prevalence and own risk to catch the disease. Comparison with socio-demographic risk factors shows poorly-calibrated beliefs with respect to age and gender. In relative terms, risk perceptions were well-calibrated: IFR was two to three times lower than expected prevalence: COVID-19 was perceived as more serious and more contagious than seasonal influenza.

### Comparative Optimism During Lockdown

**FIGURE 5:**
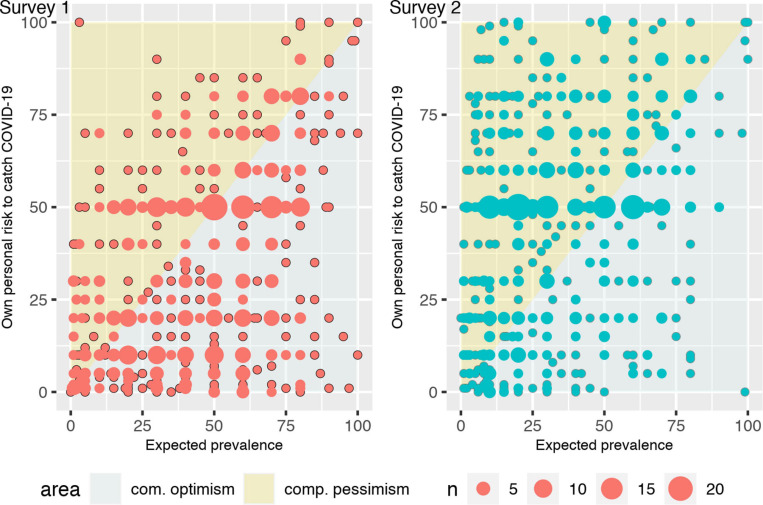
Comparative optimism in survey 1 and survey 2.

The comparison of answers to item Q2 on the assessment of own personal risk to catch COVID-19 and answers to item Q4 on the expected prevalence of the disease offers a direct measure of comparative optimism (Weinstein, 1982). Figure 5 shows the distribution of answers as a function of the survey number. During the first survey, usual findings from the literature were confirmed, notably that individuals were optimistic about their own chances to catch COVID-19: their probability to encounter the negative event was judged to be lower than others’ probability, as measured by the expected prevalence (average difference of -10.56 points, two-sided paired *t*-test, *P*-value < 0.01). The second survey shows a rather different picture, with relative pessimism as a dominant trait. At that time, respondents judged their own probability to catch the COVID-19 to be higher than the expected prevalence (average difference of 12.31 points, two-sided paired *t*-test, *P*-value < 0.01). Figure 5 shows that the reversal in comparative optimism between the two surveys was the consequence of a simultaneous increase in own personal risk to catch COVID-19 and a decrease in expected prevalence. According to the GLM on the optimism index (defined as the difference between expected prevalence and the own risk to catch COVID-19, re-scaled between 0 and 1) a large significant difference between the two surveys was found (*P*-value < 0.01).

We summarize these findings in the following observation:

H2: Comparative optimism was observed in the first survey, but it turned into comparative pessimism in the second survey. Hence, comparative optimism decreased during lockdown.

### Changes in Beliefs During Lockdown

Heterogeneity was much lower for IFRs than for the other items for which Figure 2 shows a large dispersion of the answers on the measurement scales. This difference in heterogeneity was expected due to the lower value associated to the IFRs. According to the GLM (see Table 5 in the Supplementary Material for details), no differences between the two surveys were found for the IFRs (Q1, *P* value = 0.915) and the assessment of how other people perceive their risk to catch the disease (Q3, *P* value = 0.617). For their own risk to catch COVID-19 (Q2) and the expected prevalence (Q4), we found large significant differences between surveys (both *P* values < 0.01) with an 11.5 percentage points increase in the own personal risk to be infected and an 11.1 percentage points decrease in the assessed expected prevalence.

Respondents became more pessimistic about their own risk to catch the disease and more optimistic regarding the expected prevalence. Visual inspection of Figure 2 shows a modal answer at 50 for answers to questions Q2 and Q3. Such a pattern is consistent with Bruine de Bruin et al. (2002) and might have biased the answers. Following (de Bruin et al., 2002) we fitted a beta function to each distribution to assess the importance of this “50-blip”. Table 4 shows the impact the “50s” blip on the answers to questions Q1-Q4, without correction for post-stratification. For items Q1 and Q4, it is clear from Table 4 that the “50-blip” did not operate. For item Q3, even if the percentage of excess 50s is relatively high (from 14 to 16% of the sample), the proximity of the average answer to 50 makes the possible overestimation of the risk immaterial. The same occurs for the answers to item Q2 in survey 2. The highest impact of the 50-blip was found for question Q2 in survey 1, which results in a slight overestimation of own personal risk to catch COVID-19. Removing the “50” answers in item Q2 did however not change the conclusion about significance of the difference between the two surveys (*P* value < 0.01).

**TABLE 4:** Excess% of 50s in answers to items Q1–Q4 and differences between means.

**Item**	**Survey**	**Excess%**	**Mean measured as proportion**	**Mean without 50s**	***p*-value for the difference between mean**
Q1 (IFR)	1	-0.58	0.13	0.12	0.05
	2	0.15	0.14	0.12	0.06
Q2 (own personal risk)	1	11.87	0.34	0.30	0.00
	2	17.53	0.47	0.45	0.23
Q3 (perceived risk by others)	1	14.11	0.46	0.44	0.17
	2	16.37	0.45	0.43	0.07
Q4 (expected prevalence)	1	2.17	0.45	0.44	0.39
	2	-1.85	0.34	0.32	0.09

Table 5 in the Supplementary Material shows the results of the Generalized Linear Model regressions for items Q1 to Q4 for the first survey only and the two surveys pooled together after the inclusion of control variables. A significant impact of time on reported answers was confirmed for items Q2 and Q4. As described in Section 4.2.3, being male had a strong impact on reported beliefs: men were more optimistic than women on IFR and expected prevalence. The Generalized Linear Model regressions show that the impact of gender on own risk to catch COVID-19 becomes no longer significant after controlling for socio-demographic variables, health indicators and qualitative opinions. Beliefs about others’ risk perception was not gender-specific. Age was also an important explanatory variable for self-assessment of beliefs, with elderly people being more optimistic than younger people for both IFRs, own personal risk to catch the disease and expected prevalence. Results on others’ risk perception showed a lower impact of age, except for respondents aged 18 and 19 who revealed a significantly lower assessment of others’ risk perceptions. Education appeared to be an important determinant of the assessment of IFR, with higher educational levels being associated with lower assessments of the IFR from COVID-19. The same applies to income category, with a tendency for a higher income to reduce beliefs about COVID-19. Regarding employment status, we found some evidence when both surveys were pooled together that being an employee, in either the public or the private sector, was associated with a higher assessed IFR compared to being inactive. The impact of location area was particularly important in survey 1 and almost vanished when both surveys were pooled together. A striking figure of the data is that living in a higher incidence region significantly decreased the assessment of own personal risk to catch COVID-19 and increased the assessment of others’ risk perception. Location area had however little impact on expected prevalence or predicted IFR.

Regarding health issues, no systematic pattern arose from the surveys. Having health problems was associated with decreased IFRs, but only the passage from very good to good health was significant. Personal experience with COVID-19 increased all reported answers, but it failed to reach significance. On the opposite, having relatives who had been ill from COVID-19 significantly reduced the reported IFRs. Consistently, respondents who reported a high relative risk to catch COVID-19 also reported a higher personal risk to catch the disease, an association reassuring for the quality of the data. In both surveys, they also reported higher values for others’ risk perceptions and higher expected prevalence. Respondents who were more worried about catching COVID-19 were also more likely to report high IFRs and high risk to catch the disease. No significant pattern emerged in the two other items Q3 and Q4. The perceived contagiousness of COVID-19 had a strong positive impact on reported answers in both surveys. On the opposite, the perceived seriousness of the disease never reached significance, nor did the level of confidence in reported beliefs. Finally, the duration of the epidemic was significantly associated with the expected prevalence, a result that further validates the quality of the data, and the expected IFR for the seasonal influenza was associated with higher reported levels for most items: IFR (for COVID-19), others’ perceived risk and expected prevalence.

Considered jointly, these findings are summarized in the following observation:

H3: Results shows individuals reacted to risk information: perceived own risk to catch COVID-19 increased between survey 1 and survey 2 while beliefs on how other people perceive their risk to catch the disease remained stable. IFR also remained stable between the two surveys while expected prevalence decreased. Age, gender and education -but not health-related items- were important explanatory variables for beliefs.

## Discussion and Conclusion

In this paper, we set out to investigate how people form beliefs in pandemic risk settings, by implementing two surveys in the French population just after the outbreak of COVID-19. Contrary to several previous studies on this topic, which relied on convenience sampling (Cerami et al., 2020; Wong et al., 2020), we did so using a large representative sample of the general public. Based on two successive surveys conducted during lockdown, before and after the first epidemic peak, we report self-assessments about risks including the IFR for COVID-19, the own personal risk to catch (or catch again) the disease, the risk as perceived by others and the expected prevalence rate in the French population. Our main findings were that risk perceptions in absolute terms were rather high, with the average belief reaching approximatively 16% for the IFR, i.e., three to ten times the clinical figures available at the time of the survey, and ranging from 34 to 45% for expected prevalence. Own personal risk to catch the disease increased significantly between the two surveys from 35% to 46%, whereas the perception of others’ risks remained rather stable. Compared to available evidence, such numbers show that respondents overestimated the probabilities to catch or die from COVID-19. This finding is not unusual in the literature (Ferrer and Klein, 2015) and does not necessarily reflect a strong deviation from rational behavior. As shown by Viscusi (1985), overestimating small risks fatalities rationally occurs in a Bayesian model when learning is based on partial information. In the case of the outbreak of an unknown disease, information costs are high, and one would expect that posterior Bayesian probabilities adjust slowly to their objective counterparts. While such a rational model explains why own personal risk to catch COVID-19 rose between the two surveys, it could not explain why the subjective assessment of the IFR remained stable before and after the epidemic peak.

We found mixed evidence regarding the effect of gender on the propensity to overestimate risks. In bivariate analysis, women appeared to overestimate risks more than men, but in multivariate analysis, the opposite result emerged in the first survey, while the effect was insignificant for both surveys combined. Previous evidence on this comparison is also ambiguous, with one study reporting men to be more reluctant to wear face-masks than women (Haischer et al., 2020), and another study finding mixed evidence (Howard, 2020). Concerning health protection behavior in case of pandemic outbreaks such as COVID-19, the evidence is more conclusive in that men engage less in it than women (Moran and Del Valle, 2016; Clark et al., 2020).

The theoretical part of the paper builds on the Bayesian learning model to predict a number of effects of lockdown on beliefs. In the words of Gigerenzer (2020), the Bayesian learning model is limited because it remains an as-if theory of mind. Given several sources of information and a risk assessment, the Bayesian learning model describes the optimal solution for a rational individual. One advantage of the Bayesian learning model is to make clear predictions on how experience and available information impact individuals’ risk perceptions. The model is far from being perfect, especially in the health domain. In an extensive review of medical decision-making studies, Blumenthal-Barby and Krieger (2015) found that only 10% of the studies disconfirmed the presence of a bias or heuristic in the patient population under investigation. Among the dozens of biases identified in the literature in health-related decision making, the availability heuristic and the representativeness heuristics are particularly central to risk assessment (Dawson and Arkes, 1987). Because COVID-19 was a new, unknown disease, few specific instances were available to the individual to form their judgments of likelihood. One exception is seasonal influenza. Participants to survey 1 clearly identified COVID-19 as more contagious and more dangerous than seasonal influenza. It is therefore unlikely that the availability heuristic had played a major role in risk assessment. We found large, significant, increases in the assessments of both the own risk to catch COVID-19 and expected prevalence. This shift shows individuals accounted for new information on the disease and updated their prior, hence suggesting the Bayesian learning model provided a better account of the data than the representativeness heuristic.

The Bayesian learning model has several downsides, among which the lack of explanations for the psychological processes leading to risk assessments and its inability to account for some important characteristics of ecological rationality (Marewski and Gigerenzer, 2012). One alternative is to replace Bayesian learning with fast and frugal heuristics adapted to fit risk judgments in a given decision-making context. Whereas the Bayesian learning model supposes that more information is always best, the fast and frugal heuristics can achieve superior performance when information is ignored (Hoffrage et al., 2000). Indeed, instead of weighting and averaging all sources of information as the Bayesian learning model, fast and frugal heuristics retain one single predictor, a source of information (e.g., experience) or even a selected part of a source (e.g., own personal experience with COVID-19) to update beliefs. Unfortunately, our dataset did not contain enough information to be able to identify, at the individual level, the first-order predictor of risk perceptions.

Several factors might explain why respondents had higher assessments of their own risk during lockdown and kept their appraised fatality rates constant. First, health risks, and especially IFRs, are often overestimated (Viscusi, 1991; Skinner et al., 1998; Carman and Kooreman, 2014). Second, highly publicized small risks are also often overestimated at the individual level (Lichtenstein et al., 1978; Viscusi and Hamilton, 1999). In this respect, the large media coverage of the contagiousness of the disease during lockdown could have contributed to the increase of perceived risks to catch COVID-19. In the same fashion, the epidemic peak made the risk to catch the disease more immediate, a factor that led risk perceptions to become more pessimistic (Shepperd et al., 2000). Third, risk perceptions are reflective of not only numerical information, through deliberative processes, but also of experiential factors such as the information derived from personal experiences or events that occurred in the individual’s inner circle. We found no significant evidence for an impact of personal experience with COVID-19 on beliefs, although lower risk perceptions of the IFR were reported when some of the respondent’s relatives (family members or friends) had been diagnosed with a disease. The latter result contradicts evidence described by Chen and Kaphingst (2011) in the context of lung cancer. Personal experience with health issues (health status or experience of a chronic illness) did not show a unilateral impact on beliefs: only chronic illness had a significant, and negative, impact on own risk perception to catch COVID-19.

Over and above the amount of absolute pessimism about IFRs or own personal risk to catch the disease that was found in the data, a comparison of the latter with the expected prevalence rate allowed us to study the dynamics of comparative optimism -or pessimism- during lockdown in France. Comparative optimism arises when an individual gives a comparative risk estimate that is lower than that of a relevant comparison group. The typical metric to identify comparative optimism is an average of these estimates lower than the comparison group (10, Table 1). Such a behavioral trait has been regularly found in the health domain for breast cancer and prostate cancer (Clarke et al., 2000), pneumonia or influenza (Weinstein, 1987). Answers to survey 1 confirmed this pattern with a clear indication that respondents were optimistic about their chances to catch COVID-19. However, after the epidemic peak, respondents in survey 2 revealed comparative pessimism due a concomitant increase in their perceived chances to catch the disease and a decrease in their expected prevalence rate. Such a pattern is consistent with the results of Burger and Palmer (1992) who found a decrease in comparative optimism of Californian students about natural disasters after the 1989 earthquake. One possible explanation is that the lockdown and highly pessimistic information at that time had forced the respondents to focus on information about their own vulnerability to the virus.

The survey items were based on two different question formats that might have impacted the results. The first format, corresponding to the items on the IFR and the expected prevalence, used a base population reference point to elicit beliefs. The second format, which aimed to assess own risk to catch COVID-19 and risk perception by others, used singular terms and was more open ended than the first format. We deliberately chose those two different formats because the first one explicitly refers to the distribution of a characteristic in the population (death for the IFR and diagnostic for the prevalence rate), whereas the second one was meant to emphasize features specific to the individual perspective. One concern with the use of numerical formats to express beliefs is that the answer “50” may either reflect epistemic uncertainty or an inability to translate one’s feelings into a number rather than a true numerical assessment of beliefs. As Bruine de Bruin et al. (2002) showed, this is particularly the case for items described in singular terms as compared to those items described in distribution terms. As expected in our data, more “50”s were observed with the singular terms than with the distributional terms. However, because the average answer was close to 50 anyway, we found no significant impact of the “50”s on the answer, except for the own risk to catch COVID-19 in survey 1.

The strict nationwide lockdown at the time of the two surveys imposed several constraints on data collection that might have created specific features of the data. First, due to traveling restrictions for non-essential workers, the poll institute could not organize phone surveys. Although the IFOP panel included more than 750 000 households, the usual sampling methods for representative phone surveys were not available. While this did not impact the representativeness of the data samples, it makes comparisons with traditional, phone-based, surveys on health risks conditional on the survey method. Another technical limitation was the impossibility to build a within-subject design in a longitudinal survey. As a consequence, our investigations on beliefs dynamics were entirely based on between-subject comparisons, lacking precise information away from the observed differences in average patterns.

As a conclusion, this study shows well-calibrated beliefs about COVID-19, especially when it comes to seriousness and contagiousness of the disease and to respondents’ own risk of COVID-19. While these findings were obtained in the early pandemic stage characterized with much limited knowledge of the virus and insufficient means to struggle against the outbreak (mainly, lack of medical face masks and screening tests for the general population), they pointed out the importance of eliciting and analyzing individual beliefs over time in a pandemic context where the spread of COVID-19 is highly attributable to individual risky behaviors.

## Members of the Coconel Group

Members of The COCONEL Group are: Patrick Peretti-Watel, Valérie Seror, Sébastien Cortaredona, Odile Launay, Jocelyn Raude, Pierre Verger, Caroline Alleaume, Lisa Fressard, François Beck, Stéphane Legleye, Olivier L’Haridon, and Jeremy Ward (Dohmen et al., 2011).

Aix Marseille Université, IRD, AP-HM, SSA, VITROME, Marseille, France (PP-W, VS, SC, CA, and LF); Southeastern Health Regional Observatory (ORS Paca), Marseille, France (PP-W, PV, and LF); Inserm CIC 1417, Université Paris, Faculté de Médecine Paris Descartes, AP-HP, Hôpital Cochin, Paris, France (OL); EHESP School of Public Health, Rennes, France (JR); CESP, Université Paris Sud, Faculté de Médecine UVSQ, Inserm, Université Paris-Saclay, Villejuif, France (FB and SL); Université Rennes, CNRS, CREM UMR 6211, Rennes, France (OH); and GEMASS, CNRS, Université Paris Sorbonne, Paris, France (JW).

## Data Availability Statement

The datasets analyzed for this study can be found in the Supplementary Material.

## Ethics Statement

The studies involving human participants were reviewed and approved by The ethical committee of the University Hospital Institute Méditerranée Infection (#2020-018). The patients/participants provided their written informed consent to participate in this study.

## Author Contributions

OL’H, JR, VS, and the COCONEL Group developed the survey and collected the data. OL’H analyzed the data and reported the results. AA, OL’H, and VS drafted the manuscript. All authors contributed to the article and approved the submitted version.

## Conflict of Interest

The authors declare that the research was conducted in the absence of any commercial or financial relationships that could be construed as a potential conflict of interest.
