# Supplementary material for: Beliefs and Risk Perceptions About COVID-19: Evidence From Two Successive French Representative Surveys During Lockdown
Source: Front Psychol. 2021 Feb 1;12:619145. doi: 10.3389/fpsyg.2021.619145 (PMC7882490; doi:10.3389/fpsyg.2021.619145)
Supplement: Supplementary file 1 [file Table_1.DOCX]

Supplementary Material

# *Tables 5 to 8. Generalized Linear Model estimation results*

## *Table 5: Estimation results with and without post-stratification weights for the models without covariates*

|  | Dependent variable: | | | | | | | | | |
| --- | --- | --- | --- | --- | --- | --- | --- | --- | --- | --- |
|  | Q1: IFR | | Q2: own personal risk | | Q3: others’ perception | | Q4: expected prevalence | | Comparative optimism | |
|  | With weights | Without weights | With weights | Without weights | With weights | Without weights | With weights | Without weights | With weights | Without weights |
| Survey 2 | -0.026 | 0.008 | 0.479^***^ | 0.529^***^ | -0.066 | -0.027 | -0.468^***^ | -0.470^***^ | -0.425*** | -0.460*** |
|  | (0.116) | (0.080) | (0.080) | (0.058) | (0.072) | (0.053) | (0.070) | (0.054) | (0.045) | (0.033) |
| Constant | -1.625^***^ | -1.864^***^ | -0.642^***^ | -0.667^***^ | -0.086^*^ | -0.167^***^ | -0.199^***^ | -0.215^***^ | 0.204^***^ | 0.212^***^ |
|  | (0.089) | (0.057) | (0.057) | (0.042) | (0.048) | (0.037) | (0.047) | (0.037) | (0.030) | (0.023) |
| Observations | 1,545 | 1,545 | 1,486 | 1,486 | 1,298 | 1,298 | 1,487 | 1,487 | 1,289 | 1,289 |
| Note: | ^*^p^**^p^***^p<0.01 | | | | | | | | | |

*Table 6: Estimation results with post-stratification weights*

|  | Dependent variable: | | | | | | | | | |  | |
| --- | --- | --- | --- | --- | --- | --- | --- | --- | --- | --- | --- | --- |
|  | Q1: IFR | | Q2: own personal risk | | | Q3: others’ perception | | | Q4: expected prevalence | |  | |
|  | Survey | | Survey | | | Survey | | | Survey | | | |
|  | 1 | 1&2 | 1 | 1&2 | 1 | | 1&2 | 1 | | 1&2 | |  |
| Survey 2 |  | -0.159 |  | 0.447^***^ |  | | -0.134 |  | | -0.597^***^ | |  |
|  |  | (0.130) |  | (0.101) |  | | (0.101) |  | | (0.094) | |  |
| Male | -0.392^***^ | -0.530^***^ | 0.231^*^ | 0.033 | 0.153 | | -0.008 | -0.486^***^ | | -0.346^***^ | |  |
|  | (0.137) | (0.119) | (0.123) | (0.082) | (0.106) | | (0.081) | (0.111) | | (0.076) | |  |
| Age: 30-39 | -0.204 | -0.590^***^ | -0.027 | -0.063 | 0.191 | | -0.032 | 0.017 | | -0.079 | |  |
|  | (0.184) | (0.194) | (0.169) | (0.135) | (0.185) | | (0.153) | (0.155) | | (0.124) | |  |
| 40-49 | -0.375^*^ | -0.889^***^ | -0.334^*^ | -0.102 | -0.012 | | -0.167 | -0.117 | | -0.201 | |  |
|  | (0.207) | (0.177) | (0.190) | (0.150) | (0.190) | | (0.140) | (0.171) | | (0.124) | |  |
| 50-59 | -0.162 | -1.033^***^ | -0.476^**^ | -0.452^***^ | -0.083 | | -0.177 | -0.324 | | -0.397^***^ | |  |
|  | (0.245) | (0.187) | (0.200) | (0.143) | (0.201) | | (0.149) | (0.211) | | (0.132) | |  |
| 60-69 | 0.316 | -0.841^***^ | -0.521^*^ | -0.319^*^ | -0.156 | | -0.283^*^ | -0.235 | | -0.422^**^ | |  |
|  | (0.328) | (0.250) | (0.270) | (0.171) | (0.249) | | (0.166) | (0.235) | | (0.165) | |  |
| 70+ | -0.112 | -0.607^**^ | -0.395 | -0.393^**^ | 0.103 | | -0.248 | -0.436 | | -0.679^***^ | |  |
|  | (0.332) | (0.251) | (0.327) | (0.185) | (0.253) | | (0.179) | (0.275) | | (0.166) | |  |
| Less than 19 | 0.557 | 0.185 | -0.064 | -0.302 | -1.294^***^ | | -0.754^**^ | -0.490 | | -0.364 | |  |
|  | (0.540) | (0.462) | (0.234) | (0.217) | (0.286) | | (0.370) | (0.370) | | (0.229) | |  |
| Live in couple | 0.298^*^ | -0.008 | 0.209^*^ | 0.056 | 0.006 | | 0.008 | 0.045 | | -0.014 | |  |
|  | (0.152) | (0.119) | (0.117) | (0.083) | (0.123) | | (0.092) | (0.117) | | (0.078) | |  |
| Education: high school or less than bachelor’s degree | -0.387^*^ | -1.322^***^ | 0.337^**^ | -0.008 | 0.176 | | -0.074 | 0.073 | | -0.071 | |  |
|  | (0.234) | (0.165) | (0.161) | (0.102) | (0.151) | | (0.105) | (0.166) | | (0.105) | |  |
| Bachelor’s degree or higher | -0.248 | -0.788^***^ | 0.086 | -0.0002 | 0.058 | | -0.155 | 0.085 | | -0.006 | |  |
|  | (0.169) | (0.131) | (0.136) | (0.088) | (0.121) | | (0.098) | (0.133) | | (0.086) | |  |
| Employee, private sector | 0.072 | 0.544^***^ | 0.261 | 0.210 | -0.033 | | 0.0004 | 0.056 | | -0.026 | |  |
|  | (0.188) | (0.195) | (0.210) | (0.143) | (0.186) | | (0.142) | (0.172) | | (0.120) | |  |
| Employee, public sector | 0.400 | 0.492^**^ | 0.281 | 0.259 | -0.033 | | 0.155 | 0.013 | | 0.021 | |  |
|  | (0.300) | (0.237) | (0.248) | (0.175) | (0.218) | | (0.172) | (0.219) | | (0.144) | |  |
| Self-employment | 0.118 | 0.344 | 0.213 | -0.223 | -0.131 | | -0.204 | 0.209 | | -0.219 | |  |
|  | (0.336) | (0.296) | (0.277) | (0.249) | (0.274) | | (0.196) | (0.253) | | (0.203) | |  |
| Unemployment | 0.050 | 0.190 | -0.092 | 0.218 | -0.067 | | 0.050 | 0.038 | | -0.156 | |  |
|  | (0.325) | (0.262) | (0.236) | (0.203) | (0.253) | | (0.220) | (0.232) | | (0.159) | |  |
| High income | -1.320^***^ | -0.757^***^ | -0.404^*^ | -0.067 | -0.468^***^ | | -0.137 | -0.149 | | -0.118 | |  |
|  | (0.434) | (0.171) | (0.217) | (0.114) | (0.177) | | (0.115) | (0.187) | | (0.116) | |  |
| Middle income | -0.401^**^ | -0.582^***^ | -0.133 | -0.054 | -0.106 | | -0.029 | -0.164 | | -0.156^*^ | |  |
|  | (0.173) | (0.135) | (0.160) | (0.095) | (0.139) | | (0.092) | (0.141) | | (0.093) | |  |
| Highest incidence region | 0.047 | -0.063 | -0.375^**^ | -0.043 | 0.280^**^ | | 0.057 | -0.058 | | -0.163^*^ | |  |
|  | (0.195) | (0.138) | (0.168) | (0.112) | (0.133) | | (0.094) | (0.153) | | (0.096) | |  |
| Medium incidence region | 0.125 | 0.121 | -0.268^*^ | -0.125 | 0.221^*^ | | 0.086 | -0.064 | | 0.020 | |  |
|  | (0.153) | (0.133) | (0.140) | (0.088) | (0.126) | | (0.095) | (0.129) | | (0.085) | |  |
|  |  |  |  |  |  | |  |  | |  | |  |
| General health: Good | 0.466^**^ |  | -0.029 |  | -0.020 | |  | 0.175 | |  | |  |
|  | (0.214) |  | (0.160) |  | (0.135) | |  | (0.133) | |  | |  |
| Bad or very bad | 0.367 |  | 0.414 |  | -0.352 | |  | 0.424 | |  | |  |
|  | (0.365) |  | (0.279) |  | (0.262) | |  | (0.265) | |  | |  |
| Chronic illness | 0.151 |  | -0.257^*^ |  | -0.092 | |  | -0.237 | |  | |  |
|  | (0.182) |  | (0.146) |  | (0.131) | |  | (0.151) | |  | |  |
| Has been ill from COVID-19  diagnosedYes |  | 0.453 |  | 0.521^*^ |  | | 0.531 |  | | 0.528^*^ | |  |
|  |  | (0.465) |  | (0.279) |  | | (0.394) |  | | (0.308) | |  |
| Close person has been ill from COVID-19 | -0.229 | -0.278^**^ | 0.044 | 0.076 | -0.010 | | -0.069 | -0.191 | | -0.070 | |  |
|  | (0.152) | (0.120) | (0.141) | (0.084) | (0.114) | | (0.080) | (0.130) | | (0.086) | |  |
| Relative risk to catch COVID-19 moderate | 0.109 | -0.095 | 1.092^***^ | 0.801^***^ | 0.115 | | 0.318^***^ | 0.269^*^ | | 0.164 | |  |
|  | (0.216) | (0.144) | (0.162) | (0.128) | (0.149) | | (0.118) | (0.151) | | (0.114) | |  |
| High (> 6) | -0.209 | -0.168 | 1.220^***^ | 1.195^***^ | 0.105 | | 0.345^***^ | 0.344^**^ | | 0.378^***^ | |  |
|  | (0.238) | (0.167) | (0.195) | (0.149) | (0.168) | | (0.126) | (0.172) | | (0.123) | |  |
| Worried to catch COVID-19 moderate | 0.255 | 0.091 | 0.468^**^ | 0.471^***^ | 0.082 | | 0.011 | 0.126 | | 0.016 | |  |
|  | (0.239) | (0.164) | (0.217) | (0.135) | (0.167) | | (0.123) | (0.180) | | (0.122) | |  |
| High (> 6) | 0.576^**^ | 0.458^***^ | 0.729^***^ | 0.653^***^ | 0.164 | | 0.043 | 0.259 | | 0.097 | |  |
|  | (0.244) | (0.168) | (0.222) | (0.149) | (0.148) | | (0.130) | (0.180) | | (0.125) | |  |
| Contagiousness of COVID-19: High | 0.508^**^ | 0.280 | 0.576^**^ | 0.429^***^ | 0.195 | | 0.226^*^ | 0.281 | | 0.276^**^ | |  |
|  | (0.256) | (0.188) | (0.248) | (0.126) | (0.185) | | (0.117) | (0.193) | | (0.117) | |  |
| Seriousness of COVID-19: High | 0.090 | 0.240 | -0.013 | -0.013 | -0.200 | | -0.038 | -0.029 | | 0.020 | |  |
|  | (0.214) | (0.163) | (0.179) | (0.113) | (0.173) | | (0.107) | (0.175) | | (0.106) | |  |
| Contagiousness of seasonal influenza: High | 0.059 |  | -0.039 |  | 0.065 | |  | 0.002 | |  | |  |
|  | (0.150) |  | (0.125) |  | (0.111) | |  | (0.112) | |  | |  |
| Seriousness of seasonal influenza: High | -0.265 |  | -0.055 |  | 0.001 | |  | -0.331^***^ | |  | |  |
|  | (0.164) |  | (0.140) |  | (0.109) | |  | (0.116) | |  | |  |
| Duration of the epidemic | 0.004 | 0.003 | 0.003 | 0.003 | 0.004 | | 0.004 | 0.006 | | 0.009^***^ | |  |
|  | (0.006) | (0.004) | (0.007) | (0.003) | (0.005) | | (0.003) | (0.006) | | (0.003) | |  |
| IFR for seasonal influenza | 0.046^***^ |  | 0.005 |  | 0.011^***^ | |  | 0.010^***^ | |  | |  |
|  | (0.004) |  | (0.004) |  | (0.003) | |  | (0.003) | |  | |  |
| Confidence in risk assessment: Moderate or Lower |  |  | -0.056 |  | 0.079 | |  |  | |  | |  |
|  |  |  | (0.137) |  | (0.111) | |  |  | |  | |  |
| Constant | -3.176^***^ | -0.861^**^ | -2.548^***^ | -2.228^***^ | -0.660^*^ | | -0.401^*^ | -0.565 | | -0.240 | |  |
|  | (0.533) | (0.371) | (0.359) | (0.212) | (0.376) | | (0.223) | (0.367) | | (0.202) | |  |
| Observations | 486 | 1,253 | 467 | 1,240 | 424 | | 1,083 | 462 | | 1,210 | |  |
| Note: | ^*^p<0.10 ^**^p<0.05 ^***^p<0.01 | | | | | | | | | | |  |

## *Table 7: Estimation results without post-stratification weights*

|  | Dependent variable: | | | | | | | | | | |  | |
| --- | --- | --- | --- | --- | --- | --- | --- | --- | --- | --- | --- | --- | --- |
|  | Q1: IFR | | Q2: own personal risk | | | Q3: others’ perception | |  | | Q4: expected prevalence | |  | |
|  | Survey | | Survey | | | Survey | | | | Survey | | | |
|  | 1 | 1&2 | 1 | 1&2 | 1 | | 1&2 | | 1 | | 1&2 | |  |
| Survey 2 |  | -0.166^*^ |  | 0.436^***^ |  | | -0.148^**^ | |  | | -0.619^***^ | |  |
|  |  | (0.099) |  | (0.071) |  | | (0.072) | |  | | (0.073) | |  |
| Male | -0.363^***^ | -0.406^***^ | 0.211^**^ | 0.002 | 0.203^**^ | | 0.035 | | -0.364^***^ | | -0.260^***^ | |  |
|  | (0.123) | (0.085) | (0.106) | (0.059) | (0.103) | | (0.061) | | (0.102) | | (0.061) | |  |
| Age: 30-39 | -0.339^*^ | -0.541^***^ | -0.071 | -0.071 | 0.036 | | -0.060 | | 0.007 | | 0.023 | |  |
|  | (0.198) | (0.133) | (0.173) | (0.104) | (0.168) | | (0.105) | | (0.174) | | (0.105) | |  |
| 40-49 | -0.302 | -0.679^***^ | -0.392^**^ | -0.142 | -0.225 | | -0.183^*^ | | -0.196 | | -0.099 | |  |
|  | (0.195) | (0.137) | (0.175) | (0.104) | (0.166) | | (0.105) | | (0.174) | | (0.106) | |  |
| 50-59 | -0.185 | -0.688^***^ | -0.405^**^ | -0.297^***^ | -0.341^*^ | | -0.283^***^ | | -0.350^*^ | | -0.294^***^ | |  |
|  | (0.218) | (0.141) | (0.197) | (0.108) | (0.187) | | (0.109) | | (0.193) | | (0.110) | |  |
| 60-69 | 0.030 | -0.809^***^ | -0.725^***^ | -0.282^**^ | -0.319 | | -0.218^*^ | | -0.450^*^ | | -0.339^***^ | |  |
|  | (0.278) | (0.182) | (0.249) | (0.126) | (0.227) | | (0.125) | | (0.233) | | (0.129) | |  |
| 70+ | -0.146 | -0.397^**^ | -0.493^**^ | -0.265^**^ | -0.227 | | -0.234^*^ | | -0.645^***^ | | -0.487^***^ | |  |
|  | (0.277) | (0.181) | (0.247) | (0.133) | (0.232) | | (0.133) | | (0.242) | | (0.136) | |  |
| Less than 19 | 0.378 | 0.144 | 0.091 | -0.035 | -1.185^**^ | | -0.385 | | -0.563 | | -0.229 | |  |
|  | (0.375) | (0.282) | (0.378) | (0.228) | (0.484) | | (0.242) | | (0.398) | | (0.240) | |  |
| Live in couple | 0.197 | -0.105 | 0.133 | 0.048 | 0.063 | | 0.007 | | 0.059 | | 0.003 | |  |
|  | (0.131) | (0.090) | (0.112) | (0.065) | (0.107) | | (0.066) | | (0.110) | | (0.065) | |  |
| Education: high school or less than bachelor’s degree | -0.402^**^ | -0.958^***^ | 0.311^*^ | 0.066 | 0.048 | | -0.103 | | 0.024 | | -0.053 | |  |
|  | (0.190) | (0.134) | (0.160) | (0.089) | (0.151) | | (0.091) | | (0.158) | | (0.093) | |  |
| Bachelor’s degree or higher | -0.282^*^ | -0.551^***^ | 0.142 | 0.116 | 0.015 | | -0.095 | | 0.014 | | 0.020 | |  |
|  | (0.145) | (0.098) | (0.135) | (0.074) | (0.129) | | (0.077) | | (0.132) | | (0.078) | |  |
| Employee, private sector | 0.041 | 0.271^**^ | 0.187 | 0.201^**^ | -0.0004 | | 0.037 | | -0.015 | | -0.034 | |  |
|  | (0.185) | (0.129) | (0.175) | (0.097) | (0.164) | | (0.098) | | (0.167) | | (0.098) | |  |
| Employee, public sector | 0.196 | 0.327^*^ | 0.273 | 0.246^**^ | -0.019 | | 0.154 | | 0.003 | | 0.033 | |  |
|  | (0.238) | (0.171) | (0.216) | (0.123) | (0.201) | | (0.124) | | (0.206) | | (0.125) | |  |
| Self-employment | 0.236 | 0.402^*^ | 0.159 | 0.013 | -0.069 | | -0.037 | | 0.119 | | -0.146 | |  |
|  | (0.298) | (0.209) | (0.263) | (0.156) | (0.254) | | (0.156) | | (0.249) | | (0.159) | |  |
| Unemployment | 0.058 | 0.151 | -0.246 | 0.086 | -0.235 | | -0.106 | | -0.029 | | -0.096 | |  |
|  | (0.254) | (0.177) | (0.246) | (0.139) | (0.225) | | (0.138) | | (0.232) | | (0.141) | |  |
| High income | -0.868^***^ | -0.592^***^ | -0.167 | -0.053 | -0.297^*^ | | -0.150 | | -0.088 | | -0.146 | |  |
|  | (0.215) | (0.142) | (0.173) | (0.095) | (0.162) | | (0.097) | | (0.167) | | (0.097) | |  |
| Middle income | -0.303^**^ | -0.484^***^ | 0.022 | -0.048 | 0.050 | | -0.011 | | 0.006 | | -0.124^*^ | |  |
|  | (0.138) | (0.095) | (0.128) | (0.070) | (0.122) | | (0.072) | | (0.126) | | (0.072) | |  |
| Highest incidence region | 0.071 | 0.009 | -0.303^**^ | -0.034 | 0.185 | | 0.083 | | -0.131 | | -0.106 | |  |
|  | (0.157) | (0.109) | (0.132) | (0.075) | (0.127) | | (0.078) | | (0.132) | | (0.078) | |  |
| Medium incidence region | 0.051 | 0.057 | -0.129 | 0.013 | 0.131 | | 0.069 | | -0.055 | | 0.043 | |  |
|  | (0.141) | (0.094) | (0.120) | (0.067) | (0.114) | | (0.068) | | (0.116) | | (0.068) | |  |
|  | 0.432^***^ |  | 0.123 |  | -0.057 | |  | | 0.140 | |  | |  |
| General health: Good | (0.160) |  | (0.126) |  | (0.118) | |  | | (0.123) | |  | |  |
|  | 0.511^**^ |  | 0.516^**^ |  | -0.065 | |  | | 0.374^*^ | |  | |  |
| Bad or very bad | (0.257) |  | (0.220) |  | (0.212) | |  | | (0.216) | |  | |  |
|  | -0.051 |  | -0.224^*^ |  | -0.116 | |  | | -0.143 | |  | |  |
| Chronic illness | (0.147) |  | (0.125) |  | (0.120) | |  | | (0.124) | |  | |  |
|  |  | 0.316 |  | 0.127 |  | | 0.542^**^ | |  | | 0.382^*^ | |  |
| Has been ill from COVID-19  diagnosedYes |  | (0.305) |  | (0.228) |  | | (0.236) | |  | | (0.222) | |  |
|  | -0.259^*^ | -0.229^**^ | 0.005 | 0.078 | 0.122 | | 0.070 | | -0.014 | | 0.036 | |  |
| Close person has been ill from COVID-19 | (0.139) | (0.095) | (0.113) | (0.064) | (0.109) | | (0.066) | | (0.110) | | (0.066) | |  |
|  | 0.108 | -0.023 | 1.009^***^ | 0.931^***^ | 0.062 | | 0.210^**^ | | 0.129 | | 0.152^*^ | |  |
| Relative risk to catch COVID-19 moderate | (0.170) | (0.124) | (0.156) | (0.091) | (0.133) | | (0.087) | | (0.136) | | (0.087) | |  |
|  | -0.099 | -0.022 | 1.212^***^ | 1.399^***^ | 0.144 | | 0.312^***^ | | 0.236 | | 0.349^***^ | |  |
| High (> 6) | (0.185) | (0.132) | (0.168) | (0.096) | (0.149) | | (0.093) | | (0.150) | | (0.093) | |  |
|  | 0.232 | 0.100 | 0.330^*^ | 0.296^***^ | -0.074 | | -0.007 | | -0.012 | | -0.034 | |  |
| Worried to catch COVID-19 moderate | (0.213) | (0.142) | (0.178) | (0.097) | (0.153) | | (0.095) | | (0.159) | | (0.096) | |  |
|  | 0.592^***^ | 0.427^***^ | 0.610^***^ | 0.474^***^ | 0.006 | | 0.001 | | 0.275^*^ | | 0.063 | |  |
| High (> 6) | (0.217) | (0.144) | (0.183) | (0.099) | (0.158) | | (0.097) | | (0.164) | | (0.099) | |  |
|  | 0.193 | 0.056 | 0.513^***^ | 0.380^***^ | 0.371^**^ | | 0.276^***^ | | 0.425^**^ | | 0.315^***^ | |  |
| Contagiousness of COVID-19: High | (0.208) | (0.131) | (0.180) | (0.089) | (0.162) | | (0.089) | | (0.171) | | (0.094) | |  |
|  | 0.205 | 0.327^**^ | -0.067 | -0.052 | -0.314^**^ | | -0.140 | | -0.093 | | -0.035 | |  |
| Seriousness of COVID-19: High | (0.199) | (0.133) | (0.157) | (0.085) | (0.146) | | (0.086) | | (0.148) | | (0.087) | |  |
|  | 0.055 |  | 0.022 |  | 0.046 | |  | | -0.010 | |  | |  |
| Contagiousness of seasonal influenza: High | (0.134) |  | (0.111) |  | (0.106) | |  | | (0.110) | |  | |  |
|  | -0.278^**^ |  | -0.195^*^ |  | 0.006 | |  | | -0.169 | |  | |  |
| Seriousness of seasonal influenza: High | (0.139) |  | (0.116) |  | (0.110) | |  | | (0.114) | |  | |  |
|  | 0.006 | 0.006^**^ | 0.005 | 0.006^***^ | 0.008^*^ | | 0.005^**^ | | 0.009^*^ | | 0.009^***^ | |  |
| Duration of the epidemic | (0.006) | (0.003) | (0.005) | (0.002) | (0.005) | | (0.002) | | (0.005) | | (0.002) | |  |
|  | 0.047^***^ |  | 0.005^*^ |  | 0.007^**^ | |  | | 0.007^**^ | |  | |  |
| IFR for seasonal influenza | (0.003) |  | (0.003) |  | (0.003) | |  | | (0.003) | |  | |  |
|  |  |  | 0.122 |  | 0.133 | |  | |  | |  | |  |
| Confidence in risk assessment: Moderate or Lower |  |  | (0.111) |  | (0.105) | |  | |  | |  | |  |
|  | -2.820^***^ | -1.079^***^ | -2.593^***^ | -2.347^***^ | -0.594^**^ | | -0.405^**^ | | -0.603^*^ | | -0.413^**^ | |  |
| Constant | (0.385) | (0.228) | (0.337) | (0.172) | (0.295) | | (0.165) | | (0.310) | | (0.170) | |  |
|  | 486 | 1,253 | 467 | 1,240 | 424 | | 1,083 | | 462 | | 1,210 | |  |
| Observations |  | -0.166^*^ |  | 0.436^***^ |  | | -0.148^**^ | |  | | -0.619^***^ | |  |
| Note: | ^*^p<0.10 ^**^p<0.05 ^***^p<0.01 | | | | | | | | | | | |  |

*Table 8: Generalized Linear Model regressions for items Q1 to Q4, average marginal effects, standard errors in parentheses*

|  | | ^Dependent variable:^ | | | | | | | |  |
| --- | --- | --- | --- | --- | --- | --- | --- | --- | --- | --- |
|  | | Q1: IFR | | Q2: own personal risk | | Q3: others’ perception | | Q4: expected prevalence | |  |
|  | | Survey  Survey | | Survey  Survey | | Survey  Survey | | Survey  Survey | |  |
|  | | 1 | 1&2 | 1 | 1&2 | 1 | 1&2 | 1 | 1&2 |  |
| Survey 2 | |  | -0.02 |  | 0.098*** |  | -0.033 |  | -0.135*** |  |
|  | |  | (0.016) |  | (0.022) |  | (0.025) |  | (0.021) |  |
| Male | | -0.042*** | -0.066*** | 0.047* | 0.007 | 0.037 | -0.002 | -0.114*** | -0.079*** |  |
|  | | (0.015) | (0.015) | (0.025) | (0.018) | (0.025) | (0.02) | (0.026) | (0.017) |  |
| Age category | |  |  |  |  |  |  |  |  |  |
| 30-39 | | -0.022 | -0.089*** | -0.006 | -0.014 | 0.046 | -0.008 | 0.004 | -0.019 |  |
|  | | (0.02) | (0.029) | (0.037) | (0.03) | (0.045) | (0.038) | (0.037) | (0.029) |  |
| 40-49 | | -0.039* | -0.124*** | -0.071* | -0.023 | -0.003 | -0.041 | -0.028 | -0.047 |  |
|  | | (0.021) | (0.026) | (0.04) | (0.033) | (0.046) | (0.035) | (0.04) | (0.029) |  |
| 50-59 | | -0.018 | -0.139*** | -0.099** | -0.099*** | -0.02 | -0.044 | -0.076 | -0.092*** |  |
|  | | (0.026) | (0.026) | (0.041) | (0.031) | (0.049) | (0.037) | (0.049) | (0.03) |  |
| 60-69 | | 0.038 | -0.119*** | -0.108** | -0.07* | -0.038 | -0.069* | -0.055 | -0.097*** |  |
|  | | (0.041) | (0.033) | (0.054) | (0.037) | (0.06) | (0.04) | (0.055) | (0.037) |  |
| 70+ | | -0.012 | -0.091** | -0.083 | -0.086** | 0.025 | -0.061 | -0.101 | -0.152*** |  |
|  | | (0.036) | (0.036) | (0.067) | (0.04) | (0.061) | (0.044) | (0.063) | (0.036) |  |
| Less than 19 | | 0.071 | 0.033 | -0.014 | -0.067 | -0.271*** | -0.178** | -0.113 | -0.084 |  |
|  | | (0.076) | (0.084) | (0.051) | (0.047) | (0.053) | (0.081) | (0.082) | (0.052) |  |
| Live in couple | | 0.031** | -0.001 | 0.043* | 0.012 | 0.001 | 0.002 | 0.01 | -0.003 |  |
|  | | (0.016) | (0.015) | (0.024) | (0.018) | (0.029) | (0.022) | (0.027) | (0.018) |  |
| Education | |  |  |  |  |  |  |  |  |  |
| High school or less than bachelor’s degree | | -0.027 | -0.104*** | 0.017 | -4e-05 | 0.014 | -0.038 | 0.02 | -0.001 |  |
|  | | (0.019) | (0.018) | (0.027) | (0.019) | (0.029) | (0.024) | (0.031) | (0.019) |  |
| Bachelor’s degree or higher | | -0.041* | -0.15*** | 0.07** | -0.002 | 0.042 | -0.018 | 0.017 | -0.016 |  |
|  | | (0.024) | (0.018) | (0.033) | (0.022) | (0.036) | (0.026) | (0.038) | (0.024) |  |
| Employment status | |  |  |  |  |  |  |  |  |  |
| Employee, private sector | | 0.008 | 0.067*** | 0.054 | 0.046 | -0.008 | 1e-04 | 0.013 | -0.006 |  |
|  | | (0.02) | (0.023) | (0.043) | (0.031) | (0.045) | (0.035) | (0.04) | (0.027) |  |
| Employee, public sector | | 0.045 | 0.059** | 0.058 | 0.057 | -0.008 | 0.038 | 0.003 | 0.005 |  |
|  | | (0.035) | (0.029) | (0.051) | (0.039) | (0.052) | (0.042) | (0.05) | (0.033) |  |
| Self-employment | | 0.012 | 0.04 | 0.043 | -0.047 | -0.031 | -0.049 | 0.049 | -0.049 |  |
|  | | (0.036) | (0.036) | (0.057) | (0.052) | (0.065) | (0.047) | (0.059) | (0.044) |  |
| Unemployment | | 0.005 | 0.021 | -0.018 | 0.048 | -0.016 | 0.012 | 0.009 | -0.035 |  |
|  | | (0.034) | (0.03) | (0.046) | (0.045) | (0.061) | (0.054) | (0.053) | (0.035) |  |
| Income | |  |  |  |  |  |  |  |  |  |
| Middle income | | -0.047** | -0.076*** | -0.028 | -0.012 | -0.025 | -0.007 | -0.038 | -0.035* |  |
|  | | (0.021) | (0.018) | (0.034) | (0.021) | (0.033) | (0.023) | (0.033) | (0.021) |  |
| High income | | -0.122*** | -0.093*** | -0.082* | -0.015 | -0.111*** | -0.033 | -0.035 | -0.027 |  |
|  | | (0.034) | (0.02) | (0.043) | (0.025) | (0.042) | (0.028) | (0.044) | (0.026) |  |
| Region | |  |  |  |  |  |  |  |  |  |
| Medium incidence region | | 0.013 | 0.015 | -0.056* | -0.027 | 0.053* | 0.021 | -0.015 | 0.005 |  |
|  | | (0.016) | (0.017) | (0.029) | (0.019) | (0.03) | (0.023) | (0.03) | (0.019) |  |
| High incidence region | | 0.005 | -0.008 | -0.077** | -0.01 | 0.067** | 0.014 | -0.013 | -0.036* |  |
|  | | (0.02) | (0.016) | (0.034) | (0.024) | (0.032) | (0.023) | (0.035) | (0.021) |  |
| Health status | |  |  |  |  |  |  |  |  |  |
| Good | | 0.047** |  | -0.006 |  | -0.005 |  | 0.04 |  |  |
|  | | (0.02) |  | (0.033) |  | (0.032) |  | (0.03) |  |  |
| Bad or very bad | | 0.036 |  | 0.088 |  | -0.084 |  | 0.098 |  |  |
|  | | (0.037) |  | (0.06) |  | (0.061) |  | (0.061) |  |  |
| Chronic illness | | 0.016 |  | -0.052* |  | -0.022 |  | -0.054 |  |  |
|  | | (0.02) |  | (0.029) |  | (0.031) |  | (0.034) |  |  |
| Has been ill from COVID-19 | |  | 0.063 |  | 0.116* |  | 0.13 |  | 0.123* |  |
|  | |  | (0.072) |  | (0.062) |  | (0.094) |  | (0.073) |  |
| Close person has been ill from COVID-19 | | -0.024 | -0.033** | 0.009 | 0.017 | -0.002 | -0.017 | -0.044 | -0.016 |  |
|  | | (0.016) | (0.014) | (0.029) | (0.018) | (0.027) | (0.019) | (0.03) | (0.019) |  |
| Relative risk to catch COVID-19 | |  |  |  |  |  |  |  |  |  |
| Moderate | | 0.012 | -0.012 | 0.201*** | 0.166*** | 0.027 | 0.077*** | 0.061* | 0.036 |  |
|  | | (0.024) | (0.019) | (0.027) | (0.024) | (0.036) | (0.028) | (0.034) | (0.025) |  |
| High | | -0.022 | -0.021 | 0.23*** | 0.258*** | 0.025 | 0.084*** | 0.079** | 0.085*** |  |
|  | | (0.025) | (0.021) | (0.035) | (0.03) | (0.04) | (0.03) | (0.039) | (0.027) |  |
| Worried to catch COVID-19 | |  |  |  |  |  |  |  |  |  |
| Moderate | | 0.024 | 0.01 | 0.089** | 0.1*** | 0.019 | 0.003 | 0.029 | 0.004 |  |
|  | | (0.022) | (0.018) | (0.04) | (0.028) | (0.04) | (0.03) | (0.041) | (0.027) |  |
| High | | 0.059** | 0.055*** | 0.144*** | 0.141*** | 0.039 | 0.011 | 0.06 | 0.022 |  |
|  | | (0.024) | (0.019) | (0.041) | (0.031) | (0.035) | (0.032) | (0.041) | (0.028) |  |
| Contagiousness of COVID-19: High | | 0.05** | 0.033 | 0.112** | 0.092*** | 0.046 | 0.055* | 0.064 | 0.061** |  |
|  | | (0.023) | (0.021) | (0.044) | (0.026) | (0.044) | (0.028) | (0.043) | (0.025) |  |
| Seriousness of COVID-19: High | | 0.01 | 0.028 | -0.003 | -0.003 | -0.048 | -0.009 | -0.007 | 0.004 |  |
|  | | (0.022) | (0.018) | (0.037) | (0.025) | (0.041) | (0.026) | (0.04) | (0.024) |  |
| Contagiousness of seasonal influenza: High | | 0.006 |  | -0.008 |  | 0.016 |  | 0.00035 |  |  |
|  | | (0.016) |  | (0.026) |  | (0.027) |  | (0.026) |  |  |
| Seriousness of seasonal influenza: High | | -0.028 |  | -0.011 |  | 0.00026 |  | -0.076*** |  |  |
|  | | (0.017) |  | (0.029) |  | (0.026) |  | (0.026) |  |  |
| Duration of the epidemic | | 0.00047 | 0.00043 | 0.001 | 0.001 | 0.001 | 0.001 | 0.001 | 0.002*** |  |
|  | | (0.001) | (0.001) | (0.001) | (0.001) | (0.001) | (0.001) | (0.001) | (0.001) |  |
| Case fatality rate for seasonal influenza | | 0.005*** |  | 0.001 |  | 0.003*** |  | 0.002*** |  |  |
|  | | (0.00041) |  | (0.001) |  | (0.001) |  | (0.001) |  |  |
| Confidence in risk assessment: Moderate or Lower | |  |  | -0.011 |  | 0.019 |  |  |  |  |
|  | |  |  | (0.028) |  | (0.026) |  |  |  |  |
| Observations | | 486 | 1,253 | 467 | 1,240 | 429 | 1,083 | 462 | 1210 |  |
| Note: | ^*^p<0.10 ^**^p<0.05 ^***^p<0.01 | | | | | | | | | |

# *The Bayesian learning model*

The Bayesian learning model assumes individuals have three risk information sources, with each source characterized by its informational content. The first source of information is the prior risk assessment. The second source is the experience of the individual. The third source is the risk information provided to the individual. Let p denote prior risk assessment, λ_0_ the informational content, q the risk assessment derived from experience, λ_1_ the informational content of experience, r the risk information and λ_2_ its informational content. If the prior follows a beta distribution, a common assumption in basic Bayesian analysis, then individuals’ risk beliefs, or risk perceptions, can be written as a weighted sum of the three sources:

$$beliefs=\frac{\lambda_{0}}{\lambda_{0}+\lambda_{1}+\lambda_{2}}p+\frac{\lambda_{1}}{\lambda_{0}+\lambda_{1}+\lambda_{2}}q+\frac{\lambda_{2}}{\lambda_{0}+\lambda_{1}+\lambda_{2}}r$$

Without risk information and in the absence of personal experience, the main driver of risk beliefs is the prior. In the Bayesian learning model, an uninformative prior suggests a risk belief centered around 50%: if anything can happen between 0 and 100%, the average assessment should be 50%. A typical case in which an informative prior applies is the assessment of others’ belief to catch COVID-19 in survey 1. At the time of survey 1, no information was available on others’ beliefs and no clear prediction existed for accumulated experience. The theoretical prediction is that the individuals share uninformative priors, with almost no experience and no public information on others’ beliefs. As a consequence, others’ beliefs to catch COVID-19 should be distributed around the 50% value. In addition, during lockdown, limited social interactions provided few experience opportunities to acquire information on others’ risk to catch COVID-19. The Bayesian learning model predicts stability in the assessment of others’ risk between the two surveys.

The second source of information is experience. In our study, the Bayesian learning model predicts experience with COVID-19 increases risk perceptions about the probability to catch COVID-19, at the individual level (own risk) and the social level (expected prevalence). For example, knowledge of close family/friends ill from COVID-19 are major determinants of own risk to catch COVID-19 and expected prevalence.

The last source of information is risk information. An important literature shows that highly publicized risk on some categories of the population during lockdown (e.g. elderly people, men) is often associated with overassessment of death risks (Slovic et al., 1982). In our study, the provision of public information to the individual increases the informational content of risk information, and triggers Bayesian updating in the sense of higher own risk perception for males and older people in both surveys.

An important aspect of the Bayesian learning model is its ability to predict the impact of new informational content on belief updating. For example, the model predicts that when the combined value of risk experience and prior risk are lower than the value of risk information, perceived risk increases with the informational content of risk information. The model predicts risk information on the impact and contagiousness of COVID-19 provided to the public during lockdown is expected to have further increased the assessment of own risk to catch COVID-19 and expected prevalence, especially for males and older people.

**Reference**

Slovic, P., Fischhoff, B., Lichtenstein, S., 1982. Why Study Risk Perception? Risk Anal. 2, 83–93.
